# Supplementary material for: Are carbon emissions trading and green financial instruments synergistic? -Comprehensive quantitative research based on content analysis
Source: PLoS One. 2024 Mar 7;19(3):e0298601. doi: 10.1371/journal.pone.0298601 (PMC10919601; doi:10.1371/journal.pone.0298601)
Supplement: S1 Data — (ZIP) [file pone.0298601.s001.zip › Data/地级市Z_F工作报告.zip!/╣π╢1⁄2/╓Θ║ú╩╨/└┤╫╘╣1⁄2╓┌║┼í╛┬φ┐╦╩2╛▌═°í┐_2017.docx]

各位代表：

　　现在，我代表珠海市人民政府，向大会作政府工作报告，请予审议，并请政协委员和其他列席人员提出意见。

　　一、过去五年工作回顾

　　市八届人大一次会议以来的五年，是珠海城市发展和地位大提升的五年。在党中央国务院、省委省政府和市委的正确领导下，在市人大和市政协的监督支持下，市政府认真贯彻党的十八大和十八届三中、四中、五中、六中全会精神，深入学习贯彻习近平总书记系列重要讲话精神，主动适应经济发展新常态，统筹推进产业、交通、城市和社会民生建设，经济社会发展取得显著成效，珠海成为全省发展势头最好的地区之一。

　　（一）创新驱动引领经济发展新常态。面对持续加大的经济下行压力，我们坚持稳增长和调结构两手抓，围绕创新驱动核心战略，制定实施“1+10”政策体系1，大力创建珠三角国家自主创新示范区，推进珠江西岸先进装备制造产业带建设，现代产业体系架构基本形成，多项指标增幅进入全省前列。地区生产总值突破2000亿元关口，从2011年的1410.34亿元增加到2016年的2200多亿元，年均增长9.4%。人均生产总值超过13万元。地方一般公共预算收入从143.41亿元增加到292.27亿元，年均增长17.1%。创新能力显著增强。高新技术企业从244家增加到788家，高新技术产品产值占规模以上工业总产值比重提升到56%。全社会研发投入占GDP比重提升到2.8%，每万人发明专利拥有量从6.5件提高到32件以上，全省第二。高新区在全国排名上升到第30位。引进和推荐入选国家“千人计划”2专家50人，省创新创业团队2个，省领军人才7人。以先进装备制造业为重点振兴实体经济，累计引进亿元以上先进装备制造业项目77个，完成工业投资超1000亿元，一批龙头项目建成投产，先进制造业和高技术制造业增加值占规模以上工业增加值比重分别达到48%和25%，高栏港区海洋工程装备基地成为国家新型工业化产业示范基地。现代服务业增加值占服务业比重提升到58%，金融业增加值年均增长18%，成为国家首批“旅游休闲示范城市”，获批创建“全域旅游示范区”。完成“十二五”节能减排任务，获评第三届全国国土资源节约集约模范市。

　　（二）自贸区领航特区改革开放再立潮头。横琴“三年大变化、五年成规模”目标全面完成，新区开发迈入自贸区时代，179项改革举措落地实施，“政府智能化监管服务新模式”3获全国自贸试验区最佳实践案例，37项创新经验纳入省66项可复制推广经验中，一批支持澳门经济适度多元发展的政策出台实施，澳门大学、澳门青年创业谷等项目建成启用，港澳投资企业超过1300家。全市重点领域和关键环节改革亮点纷呈。率先推行商事制度改革，商事主体增长79%，每千人商事主体数量突破153个。率先实行社会投资项目备案制、招投标方式核准负面清单制，开展企业投资项目直接落地改革试点，民间投资占比从26.8%提升到40%以上。国地税征管体制、“营改增”、零基预算4、国资国企、社会治理创新等改革全面铺开，多项改革走在全国前列。加强与“一带一路”沿线国家和地区的经贸合作，累计利用外资95.38亿美元。建立珠港澳合作官方机制，完成国务院交办的口岸查验机制创新试点工作，横琴口岸实现24小时通关。珠中江阳合作项目全面推进。

　　（三）大交通建设拓展城市发展新空间。围绕珠江西岸交通枢纽定位实施交通大建设，完成交通投资558.2亿元。港珠澳大桥主桥全线贯通，珠海30多年的“大桥梦”即将实现。广珠铁路、广珠城轨、机场高速、高栏港高速通车，结束了珠海不通铁路、“双港”5不通高速的历史。珠海港实现亿吨大港目标，新建成万吨级以上泊位10个。机场航线通达城市从21个发展到44个，旅客年吞吐量从179.7万人次增长到613万人次，增长241.1%。全面建立新型城镇化规划体系，“一河一带两轴两镇三港三心”6等57个宜居项目启动实施，西部生态新城起步区建设全面展开。成功创建国家生态市、国家生态园林城市和国家森林城市，入选中欧低碳生态综合试点城市和国家水生态文明建设试点城市，荣获首届中国生态文明奖等荣誉。

　　（四）基本公共服务均等化增进民生福祉。财政用于九项民生支出比例达到62.7%，累计投入969.6亿元，年均增长21.6%，民生保障水平保持在全国和全省前列。全体居民人均可支配收入达到3.98万元，年均增长10.7%。城镇新增就业22.76万人，城镇登记失业率保持在2.34%-2.26%的较低水平。超额完成保障性住房建设和棚户区改造任务，基本建成1.74万套。住房公积金缴存职工突破80万人，缴存覆盖率、个贷率均居全省第一。率先建立统一的城乡居民基本养老保险制度，通过自愿补缴方式将被征地农民等群体纳入职工养老保险。率先实现医疗保险“城乡一体、待遇均等”，参保人住院报销比例提高到90%以上。成功创建省推进教育现代化先进市，新建中小学和幼儿园23所，新增学位2.24万个，月保教费不超过1000元的普惠性幼儿园占比达到53.6%。同步推进公立医院和基层医疗卫生机构综合改革，实行药品“零差率”销售7，建立分级诊疗制度，设立西部和海岛地区医疗卫生发展专项资金，入选全国健康城市建设首批试点城市。基本建成“一院两馆”8和覆盖全市村居的基层文体设施体系，初步形成主城区“十分钟文体休闲生活圈”。积极回应社会关切，每年办成十件民生实事，城乡低保、农村五保、残疾人、孤儿等底线民生保障水平保持全国领先。新农村建设基本实现强村富农的阶段性目标，硬底化道路全通达，公交线路村村通，生活垃圾和污水处理设施基本覆盖行政村，法律顾问进村居实现全覆盖，155个村居年收入超过100万元。深化平安珠海建设，安全生产、食品药品安全形势持续稳定，公共安全满意度排名全国第六。圆满完成市内外对口帮扶和精准扶贫各阶段任务。

　　（五）行政体制改革助推政府治理现代化。大力推进简政放权，实施部门权责清单制度，压减行政审批事项129项、压减率达到47.6%，全面取消非行政许可事项，下放市级事权310项，向社会转移政府职能77项。全面启动“一门式一网式”政务服务9改革，线上线下办事大厅覆盖全部村居，行政审批事项网上全流程办理率达到99.15%。加强政府立法，提请市人大审议法规草案28部，制定政府规章33部。坚持依法行政，制定实施重大行政决策程序规定和专家咨询论证办法，市、区、镇政府全部建立法律顾问制度。健全廉政风险防控和监督机制，出台预防腐败条例实施细则和配套制度，建成政府投资工程廉情预警、大数据审计等监管服务平台。双拥、民族、宗教、侨务、海防打私、统计、城管、人防、气象、档案等工作都取得新成效。

　　五年来，我们把作风建设摆在政府工作的突出位置，认真落实中央“八项规定”精神，扎实开展党的群众路线教育实践活动、“三严三实”专题教育和“两学一做”学习教育，从严从实整治“四风”突出问题，开展“为官不为”专项整治，“三公”经费比2011年下降52%。

　　五年来，我们认真贯彻市人大及其常委会决议和决定，坚持依法定期向人大及其常委会报告工作，定期向政协通报情况。五年共办理代表建议986件、政协提案2391件。特别是代表、委员高度关注的6件议案都取得了实质性进展。污水和生活垃圾处理设施规划建设、斗门四小联围海堤达标建设议案顺利办结。市技工学校吉大和金湾校区、市妇幼保健院异地建设项目动工建设，市慢性病防治中心即将开工。养老服务政策保障机制基本建立，交通治堵加快推进。

　　各位代表！2016年是珠海历经风雨铸就彩虹的一年。面对特殊背景和经济开局困难等不利形势，我们坚决贯彻中央和省决策部署，着力保持工作的连续性，推动经济社会大局稳定和持续健康发展，实现了“十三五”良好开局。预计全年地区生产总值增长8.5%，一般公共预算收入增长12.3%，全体居民人均可支配收入增长10%。

　　一年来，我们扎实推进供给侧结构性改革10，制定实施“三去一降一补”11行动计划，及时出台“1+N”稳增长系列政策12，扭转了年初经济下滑的势头，保持了稳中有进、稳中向好的良好态势。全年为企业减税让利及办理出口退税超过200亿元，规模以上工业增加值由年初的-4.4%回升至5.8%，工业投资由-54.7%回升至8.2%，固定资产投资由-7.4%回升至6%，进出口降幅由-24.8%收窄至-3%。

　　一年来，我们坚持在继承中发展，进一步明确了产业、交通、城市“三大抓手”，高效协调解决了港珠澳大桥口岸工程、珠机城轨、金琴快线等一批建设项目存在的难题，有力推动了西部生态新区开发和香海大桥、洪鹤大桥、通用机场等重点项目全面施工，中海福陆海工、国机机器人等27个先进装备制造项目开工建设，格力智能装备一期等13个项目建成投产。

　　一年来，我们围绕激发发展活力，以更大的力度推进改革开放。设立城市发展投资基金，推广应用政府和社会资本合作模式，吸引社会资本参与基础设施和公共服务项目建设，推动前山河广昌村整治改造等项目取得了实质性进展。全面打造阳光法治服务政府，荣获第四届“中国法治政府奖”。深入推进珠港澳合作发展，建立起更加顺畅的沟通渠道，澳门机动车入出横琴政策落地实施，电子口岸暨国际贸易“单一窗口”13平台正式启用。提升对接港澳和服务周边城市的能力，新开通直达上海、贵阳、长沙的长途高铁，珠海正朝着珠江西岸高铁枢纽迈进。

　　一年来，我们着力解决了一批群众反映的突出问题。放宽简化了人才入户和亲属投靠落户政策。启动了一批公办中小学和幼儿园建设。家庭病床费用纳入医保支付范围，市民在家治疗也能医保报销。优化产、儿科等重点专业卫生资源配置，积极应对“二孩潮”。果断处置了西坑尾区域垃圾处理臭气扰民问题。全面调整简化旧村、旧城改造更新政策和程序。采取了一系列措施防范房地产风险，有效促进了房地产市场平稳健康发展。

　　一年来，我们以重大城市活动为载体，进一步提升了珠海的国际形象和美誉度。成功举办了第十一届中国国际航空航天博览会、第三届中国国际马戏节、珠海WTA超级精英赛14、全国帆船锦标赛等一系列重大活动，特别是坚持“以人为本、换位思考、注重细节”的理念，办出了一届交通顺畅、安全有序、精彩纷呈的航展。珠海航展已成为我国展示综合实力的重要窗口、中外军贸合作交流的大平台、珠海最夺目的城市名片。

　　各位代表，过去五年，是珠海适应把握引领新常态、迈上经济发展新台阶的五年，是大交通格局基本构建、枢纽地位日益凸显的五年，是改革开放再谱新篇、发展活力蓬勃迸发的五年，是民生投入持续加码、人民群众获得感不断增强的五年。五年的成就来之不易，是历届市委、市政府艰苦创业、接力奋进的结果，是各界群众团结一致、共同奋斗的结果，是中央和省直部门、驻珠单位、驻珠部队、港澳台同胞、海外侨胞、国际友人大力帮助、关心支持的结果。在这里，我代表市政府，向全市人民和关心支持珠海发展的各界人士，表示衷心的感谢和崇高的敬意！

　　各位代表，展望未来，我们也清醒看到，珠海还处在爬坡过坎的攻坚阶段，还面临着很多困难、竞争和挑战。一是港珠澳大桥和深中通道建设带来历史性机遇的同时，也给珠江西岸的发展格局带来深刻变化和影响。要真正成为珠江西岸核心城市和交通枢纽，我们原有的交通基础设施和城市承载力不足，如果不奋起直追，加快构建产业、交通、城市新格局，珠海有可能在新一轮区域合作与竞争中处于劣势。二是我们的经济总量还不大，辐射带动能力还有限，产业园区集聚效应不明显、配套滞后，产业创新能力、人才的总量和结构跟创新驱动的要求有较大差距。三是城市格局还没有真正展开，城乡区域发展仍不平衡，西部、海岛和农村地区基础设施、公共服务历史欠账较多。四是一些民生问题迫切需要解决，公办学位紧缺、中心城区交通拥堵、前山河整治成效不太理想等问题，群众反映比较强烈。五是制约发展的体制机制瓶颈还有不少，行政效能不高的问题还比较突出，市场活力没有充分释放，阳光法治服务政府建设任务艰巨。六是一些干部思想观念保守，改革自觉不够，担当意识不强，创新能力不足，工作落实不力。个别重大建设项目决策不够科学民主，教训深刻。我们必须直面问题，敢于担当，系统研究解决，确保取得明显改善和突破。

　　二、今后五年的目标任务

　　珠海人民的美好愿望就是政府的施政取向和奋斗目标。今后五年是珠海建设珠江西岸核心城市的关键时期，是战略优势转化为发展优势的重要窗口期。万众期待的港珠澳大桥即将通车，珠海将历史性地站在粤港澳大湾区的重要节点上；“一带一路”、自贸区、自创区和珠江西岸先进装备制造产业带建设正在深入推进，多重国家、省重大战略在珠海叠加，我们在全国、全省的战略地位愈发突出；过去几年紧锣密鼓推进的一大批重大项目，未来五年将陆续建成。珠海已经进入新一轮加快发展的战略机遇期，发展优势非常显著，全市人民对未来充满期待。我们要抓住机遇，乘势而为，推动产业、交通、城市建设全面提速，加快建设珠江西岸核心城市，努力实现经济社会更好更快发展。

　　今后五年，我市经济社会发展的主要奋斗目标是：地区生产总值突破3500亿元，城乡居民收入提前比2010年翻一番，产业、交通、城市建设取得实质性突破，率先全面建成小康社会，基本建成珠江西岸核心城市。

　　实现上述目标，必须统筹推进“五位一体”总体布局15和协调推进“四个全面”战略布局16，坚持稳中求进工作总基调，牢固树立和贯彻落实新发展理念17，坚持以提高发展质量和效益为中心，坚持以供给侧结构性改革为主线，做到“五个更加注重”，把市第八次党代会提出的战略部署落到实处：

　　——更加注重改革开放，加快建设粤港澳大湾区的桥头堡。用好特区立法权，以法治思维和法治方式引领珠海改革创新。对接国际高标准投资贸易规则，在探索粤港澳合作新模式、建设市场化法治化国际化营商环境方面，形成一批可复制推广的改革经验，把自贸区打造成联通世界的门户枢纽。系统推进行政体制、经济体制等基础性关键性领域改革，形成有利于推动经济社会持续健康发展的体制机制。坚持以开放促改革、促发展，深化珠港澳合作，建设“一带一路”战略支点，努力成为大西南对接港澳、走向海外的桥头堡，构建开放型经济新格局。

　　——更加注重创新驱动，振兴实体经济，加快建设珠江西岸区域创新中心和先进装备制造产业带核心基地。深入实施创新驱动发展战略，对接“中国制造2025”和珠江西岸先进装备制造产业带建设，扎实推进产业园区扩能增效，加快建设国家自主创新示范区和质量强市示范市，构建以先进装备制造为重点的产业大格局，形成1-2个千亿级先进制造产业集群，基本建立起以创新为引领和支撑的经济体系和发展模式。

　　——更加注重交通先行，加快建设珠江西岸交通枢纽城市。立体化推进海港、空港和高铁枢纽建设，加快建成广佛江珠城轨、珠机城轨，推进黄茅海大桥等项目，进一步打通对外联系通道。建成香海大桥、金海大桥、金琴快线、兴业路快速、洪鹤大桥等一批骨干路网，突破东西部交通瓶颈，加密主城区快速化通道。综合施策系统治理交通拥堵，构建“快-干-支-微”公交网络18，谋划推动地铁建设，建立健全智慧交通体系。推进口岸“三互”大通关19建设，建成横琴口岸及综合交通枢纽等项目，进一步提高通关便利化水平。力争通过五年努力，建成珠江西岸交通枢纽。

　　——更加注重城市提质，加快建设现代化宜居宜业城市。推进城市空间“东接西拓南进北联”20，推动主城区更新提质，高标准建成西部生态新城起步区，拉开城市格局。巩固和发展生态优势，保持环境空气质量全国领先。推进智慧城市建设，实施“互联网+政务服务”，实现信息跨部门跨层级共享共用，推动公共数据资源向社会开放。建设平安城市，推进安全发展示范市建设，提高公共安全风险预警管控和快速处置能力。

　　——更加注重共建共享，率先全面建成小康社会。持续加大民生投入，提高居民收入、公共服务和社会保障水平，补齐西部地区教育、医疗、卫生等短板。全面提升教育现代化水平，重点解决中小学和幼儿园学位紧张等突出问题。建设健康城市、卫生强市和食品安全城市。创建国家体育服务体系示范区、全民健身示范市和省公共文化服务示范项目。着力解决养老问题，建成以居家为基础、社区为依托、机构为补充、医养结合的现代养老服务体系。扎实推进新农村建设，建成全省一流、全国领先的综合性新农村发展示范区。精心做好东西部扶贫协作、对口支援和省内精准扶贫工作。

　　三、2017年工作安排

　　2017年是新一届政府的开局之年。政府工作的总体要求是：全面贯彻党的十八大以来中央各项决策部署、习近平总书记系列重要讲话精神，按照省委省政府和市第八次党代会部署要求，坚持稳中求进工作总基调，牢固树立和贯彻落实新发展理念，适应把握引领经济发展新常态，坚持以提高发展质量和效益为中心，坚持以推进供给侧结构性改革为主线，围绕建设珠江西岸核心城市目标，突出产业、交通、城市“三大抓手”，统筹稳增长、促改革、调结构、惠民生、防风险各项工作，着力构建创新型经济格局，提升开放型经济水平，振兴实体经济，增进民生福祉，努力实现经济持续健康发展和社会和谐稳定。

　　今年经济社会发展的预期目标是：地区生产总值增长8%-8.5%；规模以上工业增加值增长8%；社会消费品零售总额增长12%；固定资产投资增长15%；外贸进出口保持平稳；一般公共预算收入增长10%；全体居民人均可支配收入增长8.5%；居民消费价格涨幅、城镇登记失业率分别控制在3%和3.2%以内；节能减排降碳、高技术制造业增加值占规模以上工业增加值比重、制造业投资占全社会固定资产投资比重、生产性服务业增加值占服务业增加值比重、一般贸易进出口占外贸进出口比重等指标完成省下达目标。

　　围绕全年目标任务，今年集中力量做好八项工作：

　　（一）全面深化改革开放。

　　改革开放是特区的立身之本、活力之源。没有改革开放，就没有珠海的今天；离开改革开放，珠海就失去了发展的动力和源泉。我们一定要弘扬特区精神，在新一轮高水平改革开放大潮中攻坚克难、敢闯敢试、走在前列。

　　聚焦营造良好营商环境，以经济体制改革为重点，加大重点领域和关键环节改革攻坚力度，打造市场化法治化国际化营商环境新高地。深化商事制度改革，优化“一照一码”登记全程电子化服务系统，全面实施商事主体登记注册“同城通办”21。建立“互联网+”信用监管体系，出台商事主体信用约束和风险监管措施。全面落实“双随机、一公开”22监管，推动跨部门联合执法检查。加强市场监管，引导市场主体规范经营，促进公平税负。完善投融资机制，精简投资项目审批流程，推进企业投资项目直接落地改革，推广政府和社会资本合作模式，发挥城市发展投资基金撬动作用。加强政府投资项目储备和动态管理，争取一批项目纳入国家政策性长期低息贷款。探索建立以普惠性财税政策支持为主的产业激励机制，促进各类市场主体平等竞争、共同发展。分类推进国资国企改革，优化国有资本布局，鼓励和引导民营企业参与国企混合所有制改革。实施企业“十百千”工程23，推进大型骨干企业培育和“小升规”24、“幼狮计划”25等行动。优化民营经济发展环境，落实支持民营经济发展的各项政策措施，提高政策的可操作性和便利性，支持民营企业做大做强。全面清理涉企收费、中介收费，实行收费清单管理，进一步降低企业负担。

　　加快横琴自贸试验片区建设。对标国际高标准投资贸易规则，深化投资管理制度和贸易监管制度创新，健全以清单管理为重点的事中事后监管制度。进一步放开港澳服务业市场准入，推进粤澳信息港建设，推动粤港澳服务贸易自由化。争取自贸片区扩容，推动横琴自贸片区、保税区、十字门北片区、南湾片区、洪湾片区一体化发展。

　　加强以珠港澳合作为重点的区域合作。加快粤澳合作产业园项目落地，推进横琴澳门青年创业谷等合作项目。推动自创区与港澳科技园区共享研发资源，探索珠港、珠澳联合资助研发项目模式。落实珠港澳旅游合作协议，申报横琴国际休闲旅游岛，争取旅游资源共享政策落地，支持澳门建设“一中心一平台”26。推进横琴口岸综合交通枢纽、澳门轻轨延伸横琴线等项目建设，配合协调港珠澳大桥口岸和粤澳新通道建设，争取国家支持完善相关功能设施。改造提升口岸设施，整合现有口岸资源，完善国际贸易“单一窗口”和进出口公共技术服务平台，探索“合作查验、一次放行”等新型通关模式。推进海岛开放、水域开放和港澳游艇便利进出。落实珠中江阳一体化合作协议，做好东西部扶贫协作、对口支援和省内精准扶贫工作。

　　密切与“一带一路”沿线国家、地区的合作。搭建内地与“一带一路”沿线国家、地区的国际贸易平台和通道，在货物通关、检验检疫、质量标准、电子商务等领域探索建立合作机制。推进中拉经贸合作平台和拉美综合保税枢纽仓储物流中心建设，举办中拉博览会。协同推进“川贵广-南亚”国际物流大通道项目，推动珠海港与巴基斯坦瓜达尔港合作，推动东帝汶德光工业园等对外合作园区建设，推进横琴与伊朗格什姆自贸园区旅游合作，加快申报高栏港综合保税区。

　　加快外贸转型升级。落实稳增长政策，完善外贸企业助保贷融资平台，组织企业参加大型展会，完善挂点联系企业服务机制，建设“走出去”综合服务平台。推动外贸格局从“外资企业+加工贸易”为主向“民营企业+一般贸易”并重转变，支持民营企业开拓国际市场、扩大出口。实施出口商品竞争力提升行动，推动加工贸易创新发展，支持加工贸易企业向战略性新兴产业、高端制造业和保税物流、跨境电商等生产性服务业转型。推动跨境电子商务、外贸综合服务平台、旅游购物出口及市场采购出口等新业态发展。全面实施服务贸易行动计划，加快服务贸易和服务外包平台建设。

　　（二）加快构建创新型经济格局。

　　加强创新载体建设。深入推进珠三角国家自主创新示范区建设，全面落实自创区实施方案79项具体任务。开展“双自联动”27创新试点，探索建立自贸区和自创区联动发展的体制机制。推进高新技术企业培育计划，力争高新技术企业超过900家。落实与高校的战略合作协议，建立成果导向的财政支持机制，引导和支持高校发展研究型理工类学科，推动中大天琴计划28、暨大珠海科技创新园等项目建设。推动华南理工大学现代产业创新研究院、德国人工智能研究中心等研发机构建设，新增新型研发机构5家。健全孵化器育成体系，建成珠海信息港、清华科技园二期等一批项目。发挥打印耗材、船舶及海工国家质检中心等检验检测技术平台作用，推动创新成果转化和应用。围绕集成芯片、智能机器人、生物医药等重点领域，支持企业申报国家和省重大科技专项，组织实施一批市级核心技术攻关项目。

　　营造良好创新环境，推动创新要素加速集聚。系统梳理科技、金融、人才等政策，出台更具实效性、针对性和可操作性的措施，构建系统完善的政策体系。促进科技金融深度融合，大力发展风险投资、创业投资、天使投资，促进股权投资与信贷融资联动，统筹市、区创业投资引导基金，推动一批科技型企业上市挂牌。加强知识产权保护，搭建知识产权质押融资银企对接平台，完成国家知识产权试点城市验收，发挥横琴国际知识产权交易中心作用。加强创新人才队伍建设，推出促进人才优先发展若干措施，实施高层次人才和创新创业团队支持计划，推进实施博士后培养工程，健全高技能人才培养机制。优化海外人才来珠工作流程，推进横琴港澳人才职业资格认可。

　　推进质量强市建设。全面落实质量强市三年行动计划，弘扬“工匠精神”，提升产品质量、工程质量、服务质量和环境质量。实施标准化战略，加强先进装备制造业、战略性新兴产业标准体系建设，支持企业主导或参与制订一批国际国内标准。推进品牌发展战略，修订市长质量奖评定管理办法，创建全国深海海洋工程装备制造、办公自动化和打印耗材知名品牌示范区。加快构建现代质量监管体系，力争引进一批国家和省级公共技术检测平台。严厉打击侵权假冒违法行为。

　　（三）推进产业园区扩能增效。

　　园区是实体经济的载体，园区兴则产业兴，园区强则实业旺。必须牢牢抓住园区这个主战场，强力推进园区建设、服务提升、扩能增效。

　　加强产业规划和园区建设。编制珠海现代产业规划和新一轮园区规划，出台加强园区发展建设意见，明确各类园区功能布局和产业定位。理顺园区管理体制和运行机制，成立园区建设管理统筹协调机构。研究引入专业运营机构，探索园区“规划建设招商一体化”运作。实施园区配套优化工程，设立园区建设投资引导基金，启动一批市政公用设施和公共服务配套项目，加快建设金鼎工业园主干路网等市政配套工程，建设云洲-西电感知与电讯技术联合实验室等一批园区技术服务平台。

　　着力振兴实体经济。珠海抓实体经济，核心是抓制造业，要坚持制造业立市不动摇，防止产业空心化，让企业扎根珠海、安心发展。突出发展先进装备制造业，编制实施先进装备制造产业腾飞、高技术产业提速、传统产业提升和未来产业培育“四大产业行动计划”。谋划一批项目主动对接国家战略，争取若干重大国家创新平台、重大专项落户珠海，争取把珠海重点产业、重点项目纳入国家规划布局。出台先进装备制造企业重点培育目录，鼓励民营中小制造企业向配套件和零部件生产方向发展。推动中兴智能汽车制造及研发基地、西北工业机器人减速机、运泰利智能制造、赛纳总部及信息技术研究院等39个总投资313亿元的项目动工建设。推进银隆新能源、云洲无人船研发测试基地、新兴重工装备制造等18个总投资289亿元的在建项目加快建设。争取中海福陆海工、国机机器人、烽火通信等31个总投资215亿元的项目建成投产。全力支持格力电器创新发展、做大做强，促进丽珠等生物医药企业发展壮大，推动格力智能制造装备产业园、三一海洋重工等43个已投产项目尽快达产。深入实施工业企业技改三年行动计划，力争推动50%以上的规模以上工业企业实施技术改造，鼓励现有企业增资扩产。注重精准招商、产业链招商，建设一批驻境外经贸代表处。加强龙头企业和中小微配套企业引进，建立上市公司、金融企业“两个招商情报库”，以及航空产业和装备制造招商企业目录，全力争取更多先进装备制造产业项目落户珠海。

　　加快发展以生产性服务业为重点的高端服务业。发挥港珠澳大桥及口岸的“桥头经济”作用，及早谋划一批现代物流园，策划一批适合珠海发展的现代服务业项目。推进珠港澳物流合作园首期通关中心、空港物流保税区、广铁物流园建设，开工建设万江物流码头工程，开展中油中泰物流中心等项目前期工作。推进粤港澳金融深度合作示范区建设，推动持牌金融机构落地，吸引新兴金融机构集聚发展，推动互联网金融企业规范健康发展。优化电子商务发展环境，推动广丰跨境电商产业园等项目建设。加快推进长隆二期建设，协调推动海泉湾二期、御温泉二期、岭南大地生态度假区等项目开发建设，规划打造情侣路5A级核心景观带和城市夜景工程，规划一批高端酒店用地和大型商贸服务综合体，加大旅游市场监管力度，加快建设滨海国际休闲旅游目的地。提高航展中心利用率，引进培育更多展会和航展相关产业。

　　（四）迅速掀起新一轮交通建设大会战热潮。

　　交通既与市民生活息息相关，也是城市建设、产业振兴的重要动脉。我们要把建设枢纽型交通网络作为建设珠江西岸核心城市的基础性、先导性工程，撸起袖子加油干，坚决打赢珠江西岸交通枢纽攻坚战。

　　加快推进一批重点交通项目建设。一是完善对外高快速路网。配合推动港珠澳大桥按计划通车，同步建成珠海连接线、珠海口岸与大桥主体工程。全面推进香海大桥、洪鹤大桥、鹤港高速建设，开工建设金海公路大桥、金琴快线工程，建成洪湾枢纽互通二期，开展兴业路快速通道（北段）、港湾大道改造工程前期工作。二是推进城际轨道交通建设。加快实施珠机城轨一期工程，开工建设二期工程，推动广佛江珠城轨年内开工，推进地铁前期工作。三是完善市政路网。加快建设情侣路南段主辅线，开工建设造贝路、梅界中路、新海燕桥，打通翠前北三街、翠福路等一批主城区断头路，完成吉大路、银桦路、迎宾北路北段等道路沥青罩面工程。四是推进华威路与环洲北路德溪路衔接工程、金琴快线北延段与中山东外环衔接工程等珠中跨界道路建设。

　　交通拥堵问题社会各界高度关注，在十件民生实事征集过程中，不少市民都反映这方面的问题。本届政府将全面开展中心城区公交、道路、慢行、停车、管理等各方面的整治，系统治理城市交通综合症。今年重点深化口岸地区、上冲门户、前山片区“堵点”治理，推进情侣路南段10处人行过街设施建设，完成海滨北路交景山路等主要道路交叉口挖潜改造。进一步优化公交线网，加快规划建设主干道路公交专用道，推广微循环公交29，启动建设九洲港公交枢纽站等一批公交站场。加强主干道立体交通建设，规划建设一批人行过街设施。推广应用交通大数据，建设智慧交通运行管理平台和信号协调控制系统。优化道路停车咪表规划建设。

　　提升海港、空港、铁路集疏运功能，进一步夯实珠江西岸立体交通枢纽服务辐射功能。开工建设15万吨级石化码头、黄茅海航道，推进10万吨级集装箱码头二期、15万吨级粮食码头、疏港铁路专用线二期建设，开展高栏港20万吨级主航道、高栏港海底隧道前期工作。完善提升洪湾港区。推进珠海机场升级改造和通用机场建设，开展机场综合枢纽和九洲直升机场搬迁前期工作。深化珠港澳机场合作，争取国家继续支持外籍公务机进出境并扩展到对货机开放。继续争取开通更多珠海始发终到高铁线路。

　　（五）推进城市提质升级。

　　积极稳妥推进中心城区更新改造。坚持公共利益优先和让利于民的原则，在做好规划管控的前提下，给予村组织和村民更多自主权，放开社会资本准入限制，把城市更新改造推动起来。城中旧村更新方面，开工建设银坑村、东桥村、上冲村和广昌社区政府购买棚户区改造试点项目，推进南溪村、北山村、翠微村、鸡山村前期工作。旧工业厂房更新方面，重点推动丽珠医药集团桂花北厂区等一批项目开工，加快建设优特广场商业综合体。旧城镇更新方面，编制重点区域整体更新规划，加快推进城市之心核心区改造，争取动工改造前山汇益百货及周边片区，盘活拱北口岸南区力宝、斗门深珠皇家花园等一批“烂尾楼”项目。启动拱北、吉大、狮山、翠香、香湾、南屏等片区老旧小区综合整治提升工程。加快推进香洲渔港改造搬迁、洪湾国际渔业港建设。

　　坚持绿色、生态、低碳、宜居标准，推进新区新城开发建设。高起点建设西部生态新区，完成起步区土地清理工作，全面铺开基础设施建设。推进航空新城、斗门新城、富山新城、平沙新城和西城A、B片区等主干路网建设，建成斗门西堤路南延段、平沙新城铭恩路等16条道路。规划建设金湾综合交通枢纽，推进平沙客运枢纽站建设。启动一批水系景观工程建设，基本形成生态景观格局。推进“片区中心-新镇中心-邻里中心”三级服务中心体系建设，加快建设首批公共服务设施。建设唐家片区滨海科教新城，推进情侣北路（南段）片区、珠海北站TOD项目等市政道路工程，加快后环片区一级开发，推进北区污泥处置中心、裕华聚酯公司和珠海船舶制造公司搬迁。推进十字门中央商务区开发，建成珠海中心、城市绸带二期，启动珠海国际会展中心二期，加快形成马骝洲水道北岸高端商贸服务集聚区。推进中欧低碳生态综合试点和省新型城镇化“2511”综合试点工作30，建成天沐河防洪及生态改造工程等一批海绵城市样板项目，开工建设一批综合管廊示范段。

　　保护和巩固珠海生态优势。推进一批绿化美化工程。启动全国绿化模范城创建工作，开展美丽珠海“四大行动”31和重点林业生态建设。建设一批环保设施。完善主城区污水处理系统，基本完成平沙污水厂改扩建、三灶污水处理厂二期建设，推进西部污泥处置中心和富山、新青等一批工业污水处理设施建设。推进中信环保产业园二期项目建设，开展环保生物质热电工程二期、餐厨垃圾收运处理一体化等项目前期工作。解决一批群众关心的环境问题。推进前山河“一河两涌”沿线排洪渠、排水口截污整治和主河道生态治理试点工作，力争启动生态绿堤水系工程。启动白藤湖整治提升工程。基本完成全市12条黑臭水体治理。推进垃圾分类处理。从根本上解决西坑尾区域垃圾处理问题。全面落实河长制，建立海洋生态补偿机制，完善水源保护区扶持机制。实施控制污染物排放许可制度，加强危险废物监管，开展企业环境信用评价。

　　提升城市精细化、智慧化管理水平。加快智慧城市建设步伐。实施信息基础设施建设三年行动计划，新增光缆2185公里、公众无线通信基站2232座、公共区域WLAN热点179个。建设珠海大数据中心和大数据产业园，推进智慧城市专营项目，全面落实“互联网+智慧能源”项目。筑牢城市发展安全屏障。深化公安改革，推进警务机制创新，建立健全立体化、信息化社会治安防控体系和快速反应机制。加强农村社会治安管理，完善乡村道路标识标志。推进安全生产领域改革发展，健全落实安全生产责任制，构建风险管控和隐患排查双轨预防机制，建成高栏港石化仓储区安全管理中心等项目。加大食品安全监管力度，积极创建省食品安全城市，推进国家食品安全（横琴）创新工程、小作坊集中加工区等项目。推行安全生产、食品安全责任险，完善应急指挥系统和突发事件信息发布平台，推进气象防灾减灾、风暴潮灾害监测预警中心、电网保底网架等项目建设，开展应急演练，提高防灾减灾救灾能力。深化城市管理执法体制改革，推进城市综合运行平台建设，专项整治城市违法建设和“七乱一占” 32行为。

　　促进房地产市场平稳健康发展。坚持“房子是用来住的、不是用来炒的”，防止市场投机透支珠海未来发展的美好前景。综合运用金融、土地、财税、投资、立法等手段，加强住房市场监管和整顿，健全房地产预售监管系统，建立全市楼盘信息数据库，规范开发、销售、中介等行为。建立购租并举的住房制度，鼓励发展以住房租赁为主营业务的专业化企业，出台配建保障性住房实施意见和青年大学毕业生租赁住房补贴办法，建成一批保障性住房、人才公寓和共有产权房。

　　（六）加强保障和改善民生。

　　推动社会事业改革发展。深化教育人事制度改革，完善中小学校长职级制，推进中小学教师职称制度改革。加强高层次人才引进和师资培养培训。加快推进市技工学校建设。新建20所“粤教云”33应用学校。支持民办教育发展。深化公立医院改革，优化药品和医用耗材配送机制，完善和推进分级诊疗，推动优质医疗资源下沉。深化医疗保险支付制度改革。建立完善精神卫生服务体系，开设精神卫生专科临时病区。推动市民健康信息服务系统建设。全力支持市人民医院和中大五院进军全省三十强，推进市妇幼保健院、市慢性病防治中心、市口腔医院、市第二中医院、市第五人民医院等一批项目建设。改革完善计划生育服务管理。推进珠海艺术馆、古元美术馆、科技馆等文化设施建设，开放市博物馆和规划展览馆。加大古建筑保护力度。推动社会参与公共文化体育服务，促进西部和海岛地区文化事业发展，扶持基层文体队伍发展。积极开展全民健身运动，举办市民健身运动会。办好中国国际马戏节、珠海WTA超级精英赛、环中国国际公路自行车赛（珠海站）、全国帆船帆板锦标赛、广东国际旅游文化节等重大活动。推进文明城市和文明家庭创建工作，开展核心价值观“六进”主题教育实践活动34。丰富老年人精神文化生活，健全老年教育体系。提高底线民生“兜底”保障水平，加大残疾人、孤儿、困境儿童、特困人员、失独家庭保障力度，支持斗门区启动低收入群体精准帮扶。鼓励、支持和引导社会力量开展慈善帮扶。

　　扎实做好“三农”工作。加快发展现代农业，加大科技下乡力度，推动珠海国家农业科技园、台湾农民创业园、金湾都市农业示范区建设。促进农业文化旅游融合发展，支持斗门打造珠江水系岭南乡村风情旅游示范区，提升“十里莲江”全国休闲农业与乡村旅游基地整体水平，推进九洲控股岭南乡村风情旅游示范景区和逸丰生态养生园等项目建设，引导乡村民宿规范发展。大力推进三产融合，鼓励社会资本投入农业开发，扶持新型经营主体、农业龙头企业、专业大户和农业专业合作社发展。推进农业信息化建设，拓展农产品流通渠道，推广农产品销售“互联网+”模式，推动“珠海渔都”水产品电子商务交易平台、阿里巴巴农村淘宝项目建设，新增一批农村淘宝服务站，推进快递下乡、下海岛。完成鹤洲北海堤加固达标、小林联围涝区联合泵站主体工程，开工建设黄杨河东堤、淇澳红树林海堤加固达标工程，推进斗门白藤大闸重建、白蕉联围排涝整治和乾务联围海堤达标加固工程前期工作。改造农村低压电网，推进500千伏浪白站、会同站站址和线路走廊预控。建成高标准基本农田2.07万亩。深化农村综合改革，推动斗门建设中央农办农村改革试验联系点。深化以“三权分置”35为重点的农村土地改革，基本完成农村土地承包经营权确权登记颁证工作，全面铺开农村集体建设用地（宅基地）和留用地确权工作，深入开展农村集体产权制度改革，解决2200亩农村留用地落地问题。探索农村金融服务模式创新，推进农村信用体系试点建设，扩大涉农保险试点，推动政府民生综合责任险落地。对水产养殖户购买水产价格保险和风灾保险给予40%保费补助。

　　促进海岛有序发展。科学谋划和发展海岛旅游业，加大国际品牌旅游企业引进力度，完成国家4A级旅游景区创建工作。鼓励发展现代化深海养殖和远洋渔业，加快万山国家级海洋牧场示范区和庙湾人工鱼礁示范区建设，创建国家休闲渔业示范基地。探索开展海岛产权制度改革，促进岛上土地节约集约利用。用好海岛基础设施建设补助资金，补齐海岛交通、供水、供电、通信等基础设施短板，加快建设桂山海上风电场、桂山游艇俱乐部，开工建设唐家海岛后勤补给码头、大万山岛客运码头等项目，建成东澳岛客运码头，完成外伶仃电厂搬迁，开展主岛公路成环工程、外伶仃岛供水工程等30个预备项目前期工作。开展洁净海岛大行动，实现垃圾污水处理设施全覆盖，完成主岛存量垃圾打包外运离岛处置。推进市人民医院海岛分院建设、香洲和万山教育资源共享等工作。实施海岛老人免费、居民半票乘船优惠政策。

　　（七）办好十件民生实事。

　　今年民生实事征集首次委托第三方机构专业调查民意诉求，市民反应热烈，参与度极高。在网络票选基础上，市政府扩充加码，形成了十件民生实事。

　　一是着力解决公办学位紧张和幼儿园发展问题。新建凤凰中学、梅华中学、珠峰实验学校、中山大学附属中学、第十二小学高年级部、明珠小学、容国团小学、白藤东小学、中山大学附属小学、海愉小学、北山小学、礼和小学12所中小学。对经认定的普惠性民办幼儿园给予2万元/班/年的奖励。

　　二是关心帮扶困难群体。将残疾人生活津贴提高到160元/人/月以上，重度残疾人护理费提高到200元/人/月。将城乡最低生活保障标准提高到690元/人/月。对2200名家庭经济困难的普通高中生给予2000元/人/年的生活补助，对460名学前教育困难家庭幼儿给予1500元/人/年的资助。

　　三是降低个人就医成本。医疗保险年度最高支付限额提高到72万元。基本公共卫生服务经费标准提高到55元/人/年以上。实现省内外市参保人在珠海二级以上医院住院就医即时结算。

　　四是加大保障房建设力度。新开工2700套，基本建成2100套。

　　五是新增一批文化体育设施。新建前山、北山、湾仔、红旗、井岸、唐家湾、美平7个市民艺术中心。新建10个社区体育公园。

　　六是改善公共出行条件。新增投放100辆以上新能源公交车，新建100座公交候车亭，优化调整一批公交线路。改造提升51公里自行车专用道。建成白石桥。

　　七是增加一批养老设施。建成翠香社区、湾仔社区养老服务中心和白藤街道社会福利中心。

　　八是实施生态景观工程，拓展户外休闲空间。建成1.1万亩碳汇林、10公里健康步道、20公里林荫道、40公里绿道、30个乡村绿化美化点。实施野狸岛公园景观提升工程。

　　九是保障市民食品安全。建设一批牛羊定点屠宰车间。在全市70家农贸市场和20家大型超市开展食用农产品快速检测。启动唐家水厂改造。

　　十是促进就业和创业。城镇新增就业4万人，实现就业困难人员就业2000人。组织5万人次全民技能提升培训和1万人次大学生就业创业培训。新增3家市级创业孵化基地。资助800个创业项目。

　　（八）推进阳光法治服务政府建设。

　　加强科学民主依法决策。认真落实市人大及其常委会的决议和决定，执行重大事项报告制度，自觉接受市人大、市政协的监督。落实公众参与、专家论证、风险评估、合法性审查和集体决策机制，建成决策咨询专家库。建立利益相关方、公众、专家、媒体等列席政府有关会议制度，完善重大民生决策事项民意调查机制。办好政府门户网站和官方微博，启用“珠海政声+”官方微信公众号。全面实施“七五”普法规划，强化干部法治思维。

　　加大简政放权力度。完善权责清单、负面清单等制度，取消、下放一批经济建设和社会民生领域审批事项，清理、简化审批环节。继续清理规范政府部门行政审批中介服务事项。取消行政机关与行业协会商会的主办、主管、联系和挂靠关系，支持社会组织发展并发挥作用。深化“互联网+政务服务”改革，推行“一门式一网式”政务服务，拓展手机办事应用、企业专属网页和市民个人网页应用，推出珠海12345市民服务APP。深化行政审批标准化建设，完善行政许可、公共服务事项办事指南和业务手册，推进事项标准线上线下融合应用。

　　强化廉洁政府建设。深入贯彻《关于新形势下党内政治生活的若干准则》和《中国共产党党内监督条例》，严格落实中央“八项规定”精神，巩固党的群众路线教育实践活动、“三严三实”专题教育和“两学一做”学习教育成果。加强廉政风险防控，推进审计监督全覆盖，促进权力规范、阳光运行，努力实现干部清正、政府清廉、政治清明。深入治理“为官不为”，强化行政追责。同时，要健全正向激励机制，建立容错纠错机制，鼓励创新、宽容失败，让广大干部愿干事、敢干事、能干成事。

　　各位代表！蓝图已经绘就，实干铸就辉煌。让我们紧密团结在以习近平同志为核心的党中央周围，在省委省政府和市委的坚强领导下，抢抓机遇、担当使命、干在实处、走在前列，为建设珠江西岸核心城市而努力奋斗！
